# Gene Flow Across Genus Barriers – Conjugation of *Dinoroseobacter shibae*’s 191-kb Killer Plasmid into *Phaeobacter inhibens* and AHL-mediated Expression of Type IV Secretion Systems

**DOI:** 10.3389/fmicb.2016.00742

**Published:** 2016-05-31

**Authors:** Diana Patzelt, Victoria Michael, Orsola Päuker, Matthias Ebert, Petra Tielen, Dieter Jahn, Jürgen Tomasch, Jörn Petersen, Irene Wagner-Döbler

**Affiliations:** ^1^Department of Microbiology, Microbial Communication, Helmholtz-Centre for Infection ResearchBraunschweig, Germany; ^2^Leibniz Institute DSMZ–German Collection of Microorganisms and Cell CulturesBraunschweig, Germany; ^3^Braunschweig University of TechnologyBraunschweig, Germany

**Keywords:** plasmids, conjugation, type 4 secretion system, acylated homoserine lactones, quorum sensing (QS), roseobacter group

## Abstract

*Rhodobacteraceae* harbor a conspicuous wealth of extrachromosomal replicons (ECRs) and therefore the exchange of genetic material via horizontal transfer has been supposed to be a major evolutionary driving force. Many plasmids in this group encode type IV secretion systems (T4SS) that are expected to mediate transfer of proteins and/or DNA into host cells, but no experimental evidence of either has yet been provided. *Dinoroseobacter shibae*, a species of the *Roseobacter* group within the *Rhodobacteraceae* family, contains five ECRs that are crucial for anaerobic growth, survival under starvation and the pathogenicity of this model organism. Here we tagged two syntenous but compatible RepABC-type plasmids of 191 and 126-kb size, each encoding a T4SS, with antibiotic resistance genes and demonstrated their conjugational transfer into a distantly related *Roseobacter* species, namely *Phaeobacter inhibens*. Pulsed field gel electrophoresis showed transfer of those replicons into the recipient both individually but also together documenting the efficiency of conjugation. We then studied the influence of externally added quorum sensing (QS) signals on the expression of the T4SS located on the sister plasmids. A QS deficient *D. shibae* null mutant (Δ*luxI_1_*) lacking synthesis of *N*-acyl-homoserine lactones (AHLs) was cultivated with a wide spectrum of chemically diverse long-chain AHLs. All AHLs with lengths of the acid side-chain ≥14 reverted the Δ*luxI_1_* phenotype to wild-type. Expression of the T4SS was induced up to log2 ∼3fold above wild-type level. We hypothesize that conjugation in roseobacters is QS-controlled and that the QS system may detect a wide array of long-chain AHLs at the cell surface.

## Introduction

Roseobacters are a group of *Rhodobacteraceae* which are widely distributed in marine habitats and can reach abundances up to 20 percent in polar regions or in the North Sea (Selje et al., 2004; Giebel et al., 2011) and during algae blooms (Wemheuer et al., 2014; Voget et al., 2015). They have important ecological roles in the ocean as primary surface colonizers [recently reviewed by (Dang and Lovell, 2016)] and are “master recyclers" that are consistently correlated with phytoplankton blooms, both with respect to abundance and activity, in spite of the large diversity of algae and environmental conditions encountered in those blooms (Buchan et al., 2014). Roseobacters have relatively large genomes which collectively encode biogeochemically important pathways, but only a subset of those pathways is present in any single genome (Newton et al., 2010), and the genome content of uncultivated *Roseobacter* cells determined by single cell genome sequencing differs substantially from that of their cultured counterparts (Luo et al., 2012). Both genetic drift and horizontal gene transfer have therefore been hypothesized to play an important role in their evolution (Luo et al., 2014; Luo and Moran, 2015). Horizontal gene transfer can be mediated by transformation, transduction, or conjugation. Transformation has never been observed in roseobacters and could not be achieved experimentally (Piekarski et al., 2009). The genomes of many cultivated roseobacters encode a specialized bacteriophage like particle called GTA (gene transfer agent; Lang et al., 2002) that is able to transfer small pieces of DNA (Zhao et al., 2009; McDaniel et al., 2012) and is extremely efficient in the ocean (McDaniel et al., 2010). GTA sequences have, however, not been found in some largely uncultivated lineages, e.g., DC5-80-3, CHAB-I-5, SAG-O19, NAC11-7, that together can account for up to 60% of roseobacters in surface waters of the ocean (Zhang et al., 2016). Finally, conjugation is a powerful mechanism to shift entire plasmids across species or genus barriers, but it has not been observed in this group before (Mazodier and Davies, 1991; Thomas and Nielsen, 2005).

Roseobacters carry a wealth of plasmids [the record is held by the 12 extrachromosomal elements of *Marinovum algicola* (Pradella et al., 2010)] which can encode ecologically important traits, including a complete photosynthesis gene cluster, gene clusters for the synthesis of the antibiotic tropodithietic acid, flagella, biofilms, survival under oxidative stress, and killing of algae (Pradella et al., 2004; Kalhöfer et al., 2011; Petersen et al., 2012; Thole et al., 2012; Frank et al., 2015; Soora et al., 2015; Wang et al., 2015). Individual and compatible replication modules such as the RepABC-operon, which harbors a replicase (RepC) and a partitioning system (RepAB), ensure the stable maintenance of all plasmids (Petersen et al., 2009). Conjugation of those plasmids into other species or genera could be an important mechanism for horizontal gene transfer in roseobacters.

Type IV secretion systems (T4SS) were found in half of the 12 genome sequences of roseobacters available at the time (Moran et al., 2007). They are highly homologous to the archetypical VirB/VirD4 operon of *Agrobacterium tumefaciens* (Christie et al., 2014) and might therefore function to transfer DNA and/or proteins into other bacteria or even eukaryotic hosts such as dinoflagellates (Wagner-Döbler et al., 2010; Petersen et al., 2013; Wang et al., 2015). However, such transfer has never been shown in roseobacters and therefore the function of their T4SS is unknown.

In *D. shibae*, a species of the *Roseobacter* group which was isolated from the dinoflagellate *Prorocentrum lima* (Biebl et al., 2005) two syntenous plasmids (pDSHI01, 191-kb; pDSHI03, 126-kb) are present which contain complete T4SS systems, including the *virB* gene cluster for pilus formation, the DNA relaxase VirD2 as well as the coupling protein VirD4 (Wagner-Döbler et al., 2010; Petersen et al., 2013). *D. shibae* has been shown to provide essential vitamins to microalgae in co-culture (Wagner-Döbler et al., 2010). However, during the later stage of co-cultivation with the dinoflagellate *P. minimum*, death of the algae was observed (Wang et al., 2014a). This death was dependent on the presence of the 191-kb plasmid which was therefore termed “killer-plasmid” (Wang et al., 2015). A screen for mutants of *D. shibae* that were unable to grow anaerobically using a transposon library revealed several insertion sites on the sister plasmids (Ebert et al., 2013). In addition to the importance of those mutants for understanding anaerobic growth, they provide a tool to study plasmid transfer since they are tagged by an antibiotic resistant gene.

In many Alphaproteobacteria conjugation is regulated by cell–cell communication [usually referred to as quorum sensing (QS), e.g., in *A. tumefaciens* (Lang and Faure, 2014)] and rhizobia (Ding and Hynes, 2009). QS provides bacteria with a mechanism to adjust their “behavior” to the abundance of their own kind. For the canonical LuxR/LuxI system of Proteobacteria (Fuqua et al., 1996) the essential genetic elements are a LuxR-type transcriptional regulator and a LuxI-type autoinducer synthase. In the roseobacter group the ability for QS is widespread; about 80% of all sequenced genomes contain *luxI* homologs (Cude and Buchan, 2013; Zan et al., 2014). *D. shibae* utilizes a complex communication system comprising two chromosomal *luxI/luxR* operons (*luxI_1_/luxR_1_, luxI_2_/luxR_2_*), a third synthase gene (*luxI_3_*) located on the 86-kb plasmid downstream of an autoinducer binding gene *luxB* (Wang et al., 2014b) and three additional orphan *luxR* transcriptional regulators (Wagner-Döbler et al., 2010). These QS circuits are organized in a hierarchical way (Patzelt et al., 2013). The master synthase LuxI_1_ produces two long-chain AHLs, C18-en-HSL and C18-dien-HSL, which control the expression of the two other synthase genes *luxI*_2_ and *luxI_3_*. Knock-out of *luxI_1_* resulted in a QS null mutant which did not produce detectable levels of AHLs; it had a homogenous small cell size and a faster growth rate than the wild-type; moreover, flagella biosynthesis and T4SS were strongly down-regulated. Expression of the T4SS could be restored by addition of the cognate AHLs as well as by C18-HSL, an autoinducer which was never detected in culture supernatants of *D. shibae* (Neumann et al., 2013; Patzelt et al., 2013). Thus we hypothesized that *D. shibae* might be able to respond to non-self AHLs.

Here we tested if the 191-kb and 126-kb RepABC-type plasmids of *D. shibae* carrying a T4SS can be conjugated into a distantly related *Roseobacter* species, namely *P. inhibens* DSM 17395 (Martens et al., 2006). This strain was chosen as a recipient because it has three extra-chromosomal replicons (ECRs) whose lengths differ from those of *D. shibae* and thus plasmid transfer can be visualized directly by pulse-field gel-electrophoresis (Frank et al., 2015). We then investigated if the transcription of the T4SS on those two plasmids of *D. shibae* can be induced by chemically diverse AHLs, most of which have been shown to be produced by isolates from the *Rhodobacteraceae* but not necessarily by *D. shibae*.

## Results

### Conjugation of 191 and 126-kb *D. shibae* Plasmids into *P. inhibens*

**FIGURE 1:**
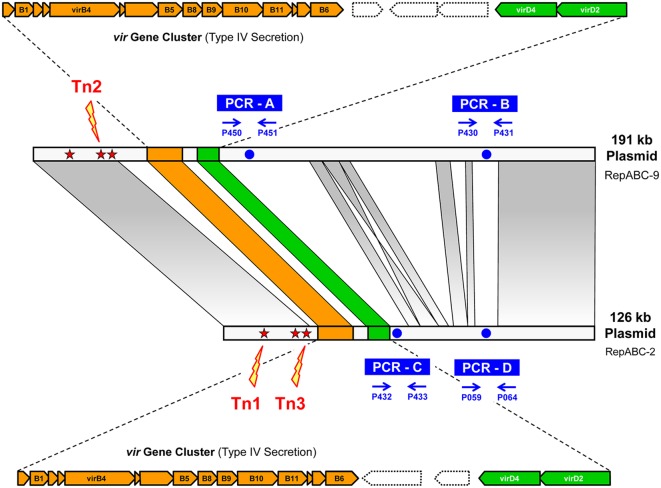
**Synteny plot of the 191 and 126-kb *repABC*-type sister plasmids of *Dinoroseobacter shibae* DSM 16493^T^ (pDSHI01, pDSHI03).** Long-range homologies with approximately identical sequences are shown with fading gray bars. The *virB* and *virD* operons of the type IV secretion system (T4SS) are highlighted in orange and green, respectively. Blue arrows indicate the localization of primer binding sites for PCR-based differentiation of the two sister plasmids. Red stars represent putative integration sites of three mariner transposons (Tn), whose precise integration site could not be determined via arbitrary PCR (Ebert et al., 2013). Yellow bolts show the actual transposon integration sites that were determined via conjugative transfer into *Phaeobacter inhibens* DSM 17395 in the current study. Tn1: Dshi_3944 (126-kb pDSHI03); Tn2: Dshi_3624 (191-kb pDSHI01); Tn3: Dshi_3964 (126-kb pDSHI03).

We used three gentamicin tagged transposon mutants of *D. shibae* as donors for conjugation. They had previously been identified based on their impaired growth under anaerobic denitrifying conditions (Ebert et al., 2013). However, the precise localization of Tn1, Tn2, and Tn3 on either the 191 or the 126-kb plasmid had not been possible, because the transposons are located in a highly conserved region of these sister plasmids (**Figure 1**; Supplementary Figure S1) and therefore the arbitrary PCR approach of O’Toole et al. (1999) that represents the standard method to determine transposon insertion sites resulted in sequences that perfectly matched with both plasmids.

The chromosome of the recipient *P. inhibens* was tagged with a kanamycin resistance gene and the conjugation mixtures of donor and recipient were spread on plates with both antibiotics. The conjugation experiment was performed twice at different times using independent cultures but similar conditions. The first experiment resulted in 228, 5, and 25 brownish colonies for Tn1, Tn2, and Tn3, respectively. The brown color is eponymous for the recipient *Phaeobacter* and correlates with the presence of its 262-kb plasmid (Petersen et al., 2011). A second independent batch of putative transconjugants was established, and four colonies from the first plus six colonies from the second batch were passaged and DNA was isolated. The authenticity of *P. inhibens* as the host of all 30 putative transconjugants was proven via PCR with specific primers for the 262-kb plasmid (Supplementary Figure S1). To detect *D. shibae* plasmids in *P. inhibens* transconjugants, four primer pairs that specifically bind to the non-syntenous parts of the two sister plasmids of *D. shibae* were designed (**Figure 1**). The PCR revealed successful conjugation of both *D. shibae* plasmids into *P. inhibens* (Supplementary Figure S1) and allowed to determine the precise integration site of the transposons *ex post* (Supplementary Figure S2). Tn1 and Tn3 were present in all 10 transconjugants of their respective conjugation mixtures and were located in the 126-kb plasmid pDSHI03. Tn1 was located in the cytochrome C biogenesis gene Dshi_3944 (YP_001542153) and Tn3 in the cation eﬄux gene Dshi_3964 (YP_001542173). Tn2 was similarly present in all 10 transconjugants of its conjugation mixture; the strong PCR signals clearly document the successful conjugation of the 191-kb plasmid pDSHI01 into *P. inhibens*. The transposon had been inserted into Dshi_3624 (YP_001541838), another gene of the cation eﬄux system. Interestingly, PCR bands for the 126-kb plasmid pDSHI03 were additionally found in transconjugant I-3 of Tn2 (Supplementary Figure S3B), indicative of the presence of both plasmids in the recipient *P. inhibens*. Two plausible scenarios would explain the transfer of the non-transposon-tagged plasmid. First, recombination of the two sister plasmids in their highly conserved large (>50-kb) syntenous regions may have resulted in a composite plasmid of 317-kb that was conjugated into *P. inhibens*. Second, under the assumption that conjugation is a very efficient process, the 126-kb plasmid pDSHI03 may simply have been transferred independently through the existing connecting pilus.

### Plasmid Profiles of Representative *P. inhibens* Transconjugants

**FIGURE 2:**
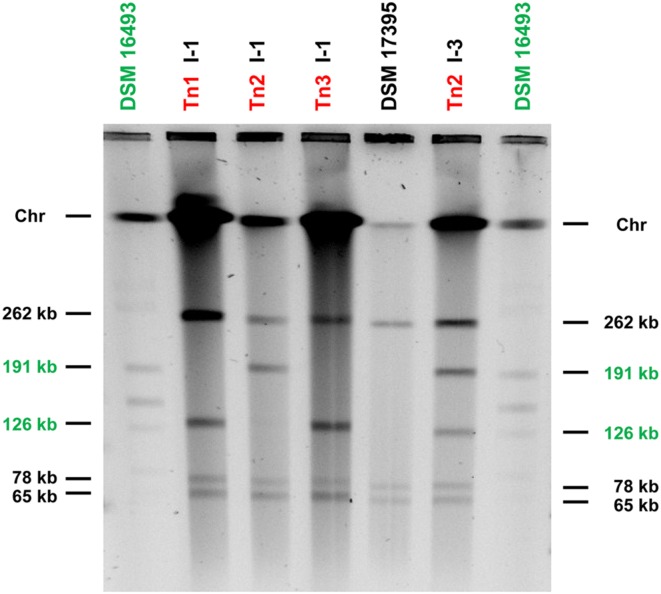
**Plasmid profiles of the *P. inhibens* DSM 17395 tranconjugants Tn1 (I-1), Tn2 (I-1), Tn3 (I-1), and Tn2 (I-3) obtained by pulsed-field gel electrophoresis (PFGE).** The PFGE conditions for separation of the high molecular weight genomic DNA were as follows: 1.0% (w/v) agarose gel with pulse times of 1–40 s for 25 h at 200 V (6 V/cm). Conjugative plasmids of the donor strain *D. shibae* DSM 16493^T^ are highlighted in green. The completely sequenced wild-type strains served as references and size standards. Chr, chromosomal DNA.

In order to differentiate between both alternatives we performed PFGE experiments and determined the plasmid pattern of representative transconjugants. The donor *D. shibae* with five plasmids (191, 154, 126, 86, and 72-kb) and the recipient *P. inhibens* with three plasmids (262, 78, and 65-kb) served as references and size standards. The conditions of PFGE were optimized to reduce the amount of plasmids with a covalently closed conformation (ccc) and to obtain linearized replicons that are easier to compare. Accordingly, all plasmids of *P. inhibens* wild-type and the four tested transconjugants are linear, whereas the faint bands above 262-kb of the *D. shibae* wild-type represent residual ccc-plasmids (**Figure 2**). The plasmid profiles illustrate the presence of an additional 126-kb plasmid in the recipient strains of Tn1 and Tn3, whereas the transconjugant Tn2 (I-1) obtained the 191-kb replicon, which is in perfect agreement with the PCR assays. Finally, the exceptional transconjugant Tn2 (I-3) clearly contains two replicons with expected sizes of 126 and 191-kb, supporting the independent transfer of both sister plasmids into *Phaeobacter* during conjugation.

### Selection of AHLs for Testing the Response of *D. shibae*

The expression of the T4SS on the sister plasmids has been shown to be down-regulated in the QS null-mutant *D. shibae* Δ*luxI_1_* (Patzelt et al., 2013). Here, we tested which AHLs were able to restore the wild-type gene expression in this strain. We used four AHLs that are produced by *D. shibae* (C14-en-HSL, 3-oxo-C14-HSL, C16-en-HSL, and C18-dien-HSL; Neumann et al., 2013; Wang et al., 2014b) and six AHLs that have never been detected in cultures of *D. shibae* (C12-HSL, C14-HSL, C16-HSL, 3-oxo-C12-HSL, 3-oxo-C14-en-HSL, and 3-oxo-C16-en-HSL). One of the main signals produced by *D. shibae* itself, C18-en-HSL, and the corresponding non-self C18-HSL were not tested, because they have already been analyzed previously (Patzelt et al., 2013). The short-chain C8-HSL served as a negative control. AHLs were added to Δ*luxI_1_* cultures in final concentrations of 0.1, 0.5, 2.5, or 5 μM.

### Long-Chain AHLs Restore the Wild-Type Growth Rate

**FIGURE 3:**
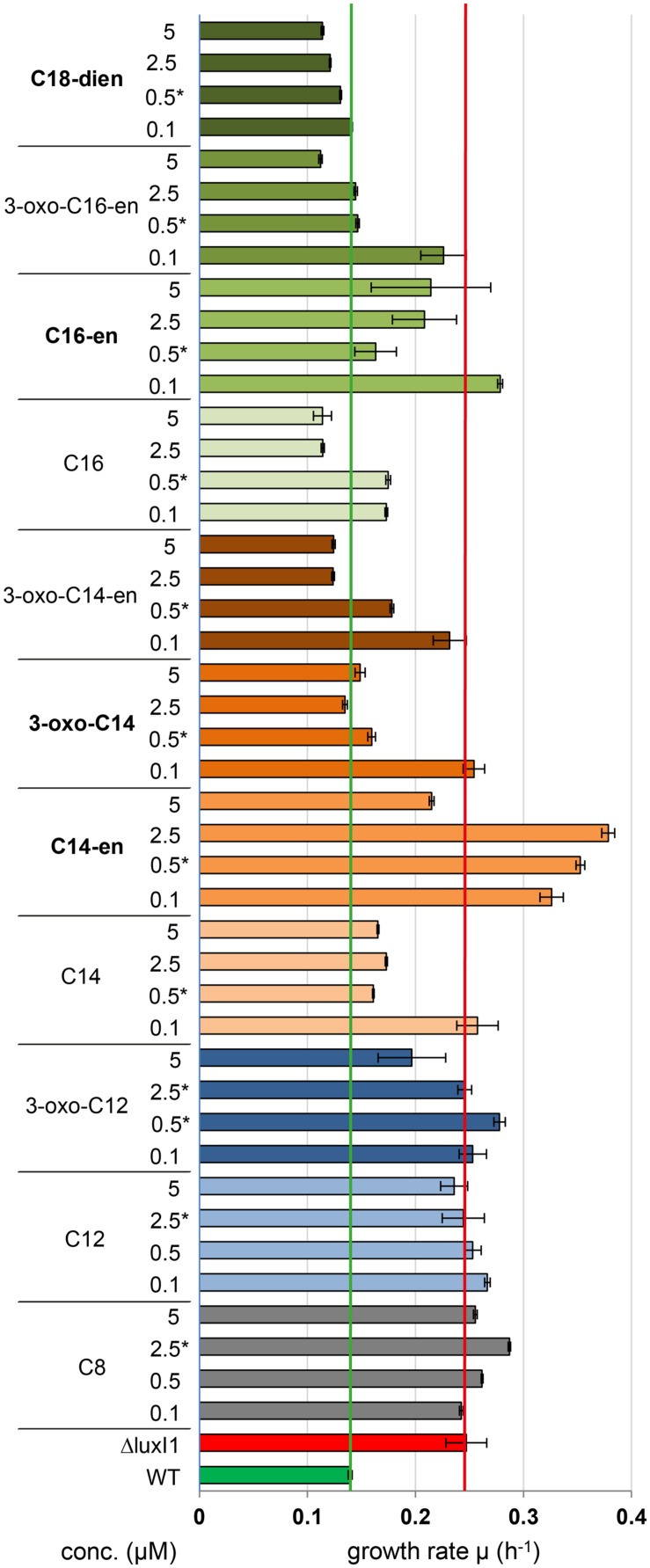
**Growth of the quorum sensing (QS) null mutant *D. shibae* Δ*luxI_1_* supplemented with external acyl-homoserine lactones AHLs.** Indigenous AHLs are shown in bold letters. Data for signal molecules with the same acyl side-chain length are displayed in the same color. Mean and standard deviation for three biological replicates are shown. Vertical lines mark the wild-type (green) and mutant (red) level. Asterisk (^∗^) labeled concentrations were used for subsequent experiments.

Previous work had shown that the QS null mutant *D. shibae* Δ*luxI_1_* grows faster, reaches a higher cell density and has a shorter lag-phase than the wild-type (Patzelt et al., 2013). Here we used high-throughput BioScreen measurements to determine the effect of AHLs on growth. **Figure 3** shows that C8-HSL and C12-HSL had no effect on the growth rate of the mutant at any tested concentration, while 3-oxo-C12-HSL caused a slight reduction of its growth rate only at the highest tested concentration of 5 μM. All other tested AHLs restored the wild-type growth rate in a concentration dependent manner. For all following experiments the lowest common active concentration of 0.5 μM was used except for C8-HSL, C12-HSL, and 3-oxo-C12-HSL where a concentration of 2.5 μM was tested in order not to miss a response.

### Long-Chain AHLs Restore Pleomorphism in the QS Null Mutant

**FIGURE 4:**
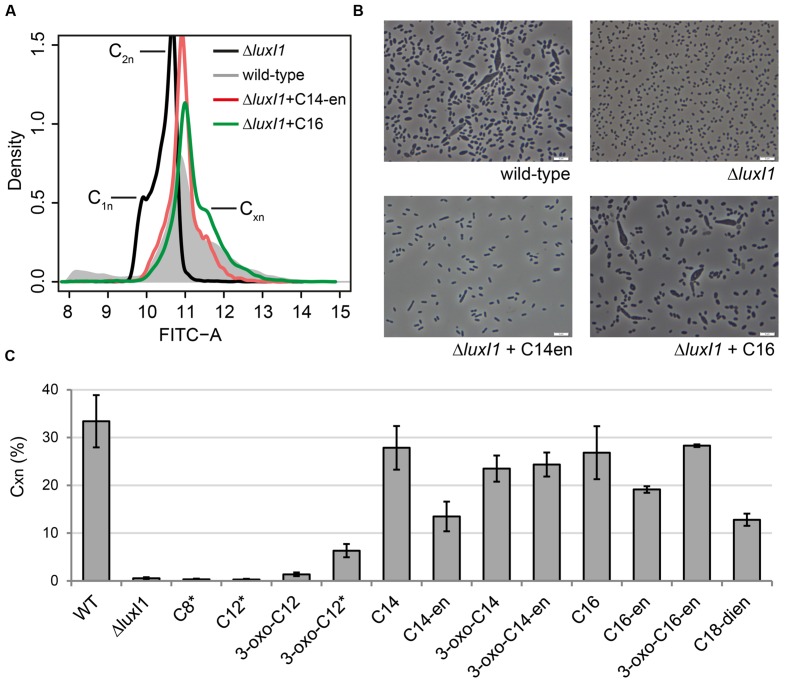
**Flow cytometric and microscopic analysis of the QS null mutant *D. shibae*Δ*luxI_1_* cultivated with externally added AHLs. (A)** Distribution of chromosome equivalents in the wild-type culture (gray), Δ*luxI_1_* (black), Δ*luxI_1_* provided with C14en (red), and Δ*luxI_1_* provided with C16-HSL (green). **(B)** Microscopic pictures of wild-type, Δ*luxI_1_* and Δ*luxI_1_* supplemented with C14en-HSL and C16-HSL. **(C)** Determination of the fraction of cells containing multiple chromosome equivalents per cell (C_xn_) at mid-exponential growth in cultures with the indicated AHLs [added at a concentration of 0.5 μM; asterisk (^∗^) indicates a concentration of 2.5 μM].

Because the growth rate of *D. shibae* is related to its mode of cell division we investigated the effect of external AHLs on chromosome content distribution and pleomorphism. Stoichiometric DNA staining and subsequent flow cytometric analysis was used to determine the percentage of cells that contained multiple chromosome equivalents (C_xn_; **Figure 4**). The QS mutant lacked cells containing more than two chromosome equivalents (**Figure 4A**) and showed uniform cell morphology (**Figure 4B**). Its DNA distribution pattern showed the re-occurrence of cells with higher fluorescence intensities when supplemented with C14-en-HSL or C16-HSL. The percentage of cells with more than one chromosome equivalents in cultures supplemented with diverse external AHLs is shown in **Figure 4C**. In the wild-type culture 33% of the cells contained multiple chromosome equivalents. This fraction was not present in the Δ*luxI_1_* mutant or in cultures provided with C8-HSL and C12-HSL, both of which had also failed to induce the slow wild-type growth. All other tested long-chain AHLs induced the occurrence of C_xn_ cells to a different extent, with C14-HSL, C16-HSL, and 3-oxo-C16-en-HSL reaching values closest to the wild-type.

### Effect of C8-HSL and C12-HSL on Gene Expression

Next we investigated the response of the Δ*luxI1* mutant to AHL treatment on the transcriptome level. Gene expression at mid-exponential growth phase of Δ*luxI1* alone and Δ*luxI1* cultivated with AHLs was compared to that of the wild-type (Dataset S1 and Supplementary Figure S3). 360 genes showed a differential gene expression, in accordance with the previous results (Patzelt et al., 2013). 47% of them encoded hypothetical proteins. In the C8-HSL and C12-HSL supplemented samples only five genes were upregulated (Supplementary Figure S1), among them Dshi_2278 which encodes a putative DMSO reductase. In those samples the AHLs had been added at 2.5 μM requiring a larger volume of the DMSO stock solution. Thus, activation of these genes is probably a response to the solvent that can be used by the cell as electron acceptor. Other than that, the transcriptome profiles of the Δ*luxI1* mutant alone and supplemented with C8-HSL and C12-HSL were similar, confirming the growth experiments.

### Effect of Long-Chain AHLs on Quorum Sensing Controlled Genes

**FIGURE 5:**
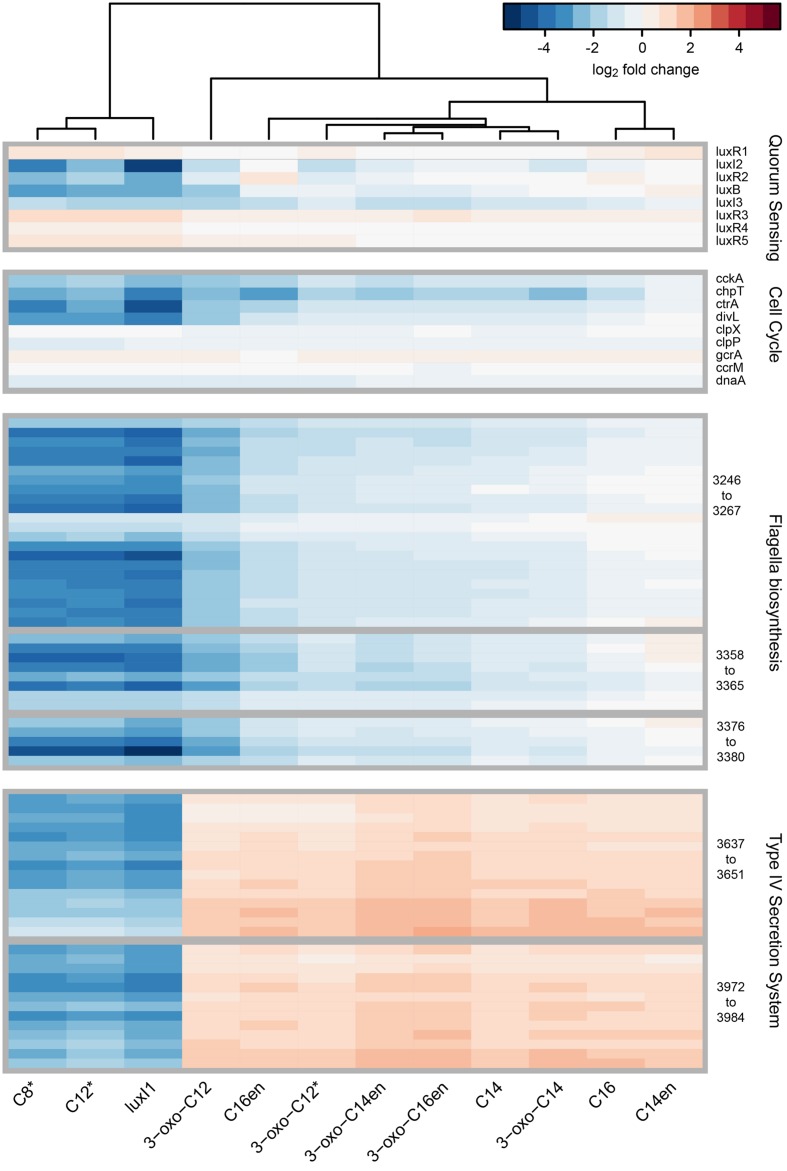
**Comparative transcriptome analysis of the *D. shibae* Δ*luxI1* mutant cultivated with different AHLs.** Heatmap visualization of log2 fold changes of QS, cell cycle, flagella, and T4SS gene expression in *D. shibae* Δ*luxI1* and Δ*luxI1* supplemented with the indicated AHLs compared to the wild-type. AHLs have been added at 0.5 μM, asterisk (^∗^) indicates a concentration of 2.5 μM. Samples were taken at the mid-exponential growth phase (OD_600_ 0.4).

We then focused on genes constituting the QS circuits of *D. shibae* (**Figure 5**). The expression of the cognate *luxR_1_* (Dshi_0311) and the orphan regulators *luxR3, luxR4*, and *luxR5* (Dshi_1550, 1815, 1819) was not significantly changed in any sample. The expression of the *luxR_2_/luxI_2_* pair (Dshi_2851, 2852) as well as of the plasmid encoded third synthase *luxI_3_* and the autoinducer binding gene *luxB* (Dshi_4067) located directly downstream could be restored to wild-type levels or slightly higher by some AHLs.

*Dinoroseobacter shibae* possesses nine homologs to the *Caulobacter crescentus* cell cycle control genes, of which four, *ctrA, cckA, chpT*, and the putative *divL* homolog were significantly down-regulated in the QS mutant. Their expression was increased to a different extend by all tested long-chain AHLs, although only C14-en-HSL restored the wild-type expression level completely. The biosynthesis machinery for the polar flagellum of *D. shibae* is encoded in three gene clusters (Dshi_3246-3268, 3358-3365, 3376-3380) which are controlled by CtrA (Wang et al., 2014b). Their expression was reduced in the QS null mutant and increased in the presence of all long-chain AHLs tested; full wild-type expression level was restored with C14-en-HSL and C16-HSL.

### Overexpression of T4SS after Addition of Long-Chain AHLs

The two plasmid encoded T4SS gene clusters (Dshi_3637-3651, 3972-85) in *D. shibae* have previously been shown to respond more strongly to AHLs than any other of the QS regulated genes (Patzelt et al., 2013). This finding can now be extended to the various long-chain self-produced and non-native AHLs tested here. All of them strongly induced the expression of the T4SS beyond wild-type level. The highest induction was observed when the mutant was supplemented with 3-oxo-C14-en-HSL and 3-oxo-C16-en-HSL.

## Discussion

Our experiments demonstrate the conjugative transfer of both the 126 and the 191-kb plasmid of *D. shibae* (pDSHI01, pDSHI03) into *P. inhibens*, suggesting that the T4SS located on them is indeed mediating conjugation as predicted by *in silico* analyses (Petersen et al., 2012). The obtained transconjugants verify that RepABC-type plasmids are stably maintained in *P. inhibens* DSM 17395 in agreement with the presence of the plasmid pInhi_B88 (RepABC-8) in the type strain *P. inhibens* DSM 16374^T^ (Dogs et al., 2013). Moreover, the establishment of both replicons in the transconjugant strainTn2 (I-3) validates the compatibility of RepABC-9 (pDSHI01) and RepABC-2 (pDSHI03) type plasmids not only in *D. shibae*, but also in *P. inhibens* (Petersen et al., 2009). However, the most important novelty of the current study is the first experimental proof of conjugation between different representatives of the *Roseobacter* group. Individual and concerted conjugation of the two sister plasmids demonstrate the efficient exchange of ECRs even between phylogenetically only distantly related mating partners such as *D. shibae* and *P. inhibens*. Thus conjugation across genus barriers may be an important mechanism for horizontal gene transfer in the *Roseobacter* group and contribute to its ecological success.

*Dinoroseobacter shibae* responded to a diverse spectrum of long-chain AHLs added to the cultivation medium; the active AHLs were chemically diverse, with acyl-side chain lengths between 14 and 18 carbon atoms, modifications at the third carbon atom, and un-saturations in the side chain. In spite of different structures of the signaling molecules, the cells responded in a very similar manner, only the strength of the response showed minor differences. This observation is not in accordance with the strict stereo-specific binding of AHLs to their cognate LuxR type transcriptional regulators. Crystal structures of TraA of *A. tumefaciens* and LasR of *Pseudomonas aeruginosa* demonstrate that their AHL is deeply buried in the binding pocket and this binding is abolished already by conserved point mutations (Bottomley et al., 2007). Our findings are also not consistent with the direct binding of the AHLs to an intracellular receptor, since the membrane is a barrier against diffusion of long-chain AHLs (Krol and Becker, 2014). Thus, the data suggest that the tested long-chain AHL signals were detected at the cell surface. Recently, is was shown that homologs of the fatty acid transporter FadL of *Escherichia coli*, which is located in the outer membrane, are found in some rhizobia and increase the sensitivity of e.g., *Sinorhizobium meliloti* to externally added long-chain AHLs significantly, most likely by importing them into the cytoplasm, where they bind their transcriptional regulator (Krol and Becker, 2014). A homolog of *fadL* is not present in *D. shibae* (unpublished results). An entirely different approach is applied by *Vibrio harveyi* and *V. cholera;* here, a phosphorylation cascade relays signal detection into the cell using two component signal transduction systems (TCS; Henke and Bassler, 2004). In this case information about the structural diversity of the detected signals is lost at the cell surface, and the transcriptional regulator is activated by phosphorylation rather than by binding of AHLs; this mechanism seems to be more in accordance with our findings. The CtrA phosporelay of *D. shibae* activates the downstream QS genes in response to the signal synthesized by the LuxI_1_ master synthase (Wang et al., 2014b) and might in fact represent a sensing mechanism for long-chain AHLs.

Induction of T4SS gene expression was always much stronger than that of the other QS controlled genes. This might be caused by the larger number of gene copies of the T4SS which are located on the two sister plasmids. Interestingly, all AHLs induced transcription of the T4SSs up to log2 threefold above the level found in the wild-type. The long-chain AHLs synthesized by LuxI_1_ in the wild-type may have to be processed or exported to naturally activate the QS signaling cascade, and this route was bypassed by external addition of AHLs. Another explanation would be that the T4SS, unlike most other QS-controlled genes, are not part of the CtrA-regulon (Wang et al., 2014b) and thus are regulated differently.

*Rhodobacteraceae*, and in particular roseobacters, produce overlapping bouquets of AHLs, rather than species specific AHLs (Wagner-Döbler et al., 2005). Molecules used in this study have been identified in *Rhodobacter capsulatus*, *R. sphaeroides*, *Roseovarius tolerans*, and *Jannaschia helgolandensis* (Schäfer et al., 2002; Wagner-Döbler et al., 2005; Cataldi et al., 2011; Bruns et al., 2013). Thus QS in roseobacters may adjust gene expression not so much to the density of the species that is producing the signal, but to the total density of AHL producing strains within the community.

In conclusion, we showed that the sister plasmids of *D. shibae* carrying T4SSs are conjugative, and that the T4SS gene clusters are upregulated by diverse long-chain AHLs. It is therefore tempting to speculate that conjugation can be triggered by AHLs in roseobacters like in other Alphaproteobacteria (Ding and Hynes, 2009; Lang and Faure, 2014). To prove this experimentally, plasmids in the *D. shibae* Δ*luxI_1_* mutant could be tagged with an antibiotic resistance gene via transposon mutagenesis. Conjugation frequencies would then have to be quantified depending on the presence, type and amount of AHLs added.

Roseobacters play important roles as colonizers of micro- and macro algal surfaces in the ocean (Buchan et al., 2014), and it has been hypothesized that horizontal gene transfer might contribute to the higher activity and adaptability of such biofilms (Dang and Lovell, 2016). The surface associated lifestyle provides both the high cell densities and cell–cell contact that are required for QS and conjugation to function efficiently. The possible link between those two physiological traits hypothesized here could therefore represent an important adaptation for survival in the ocean.

## Experimental Procedures

### Culture Conditions, Media, and AHLs

*Dinoroseobacter shibae* strains [DFL12^T^ wild-type DSM 16493^T^ (Biebl et al., 2005) and Δ*luxI_1_* (Patzelt et al., 2013)] were grown at 30°C and 160 rpm in defined sea water minimal medium supplemented with 5 mM succinate as described (Tomasch et al., 2011). Pre-cultures were inoculated from fresh half-concentrated Marine Broth (MB) agar plates (MB, Difco 2216) and grown over night before they were transferred to fresh minimal medium. For cultivation of Δ*luxI_1_* the agar was supplemented with 150 μg/ml gentamicin. AHLs were purchased from Cayman Chemicals (Ann Arbor, MI, USA) and prepared as 1 mM stocks in DMSO. AHLs were added to Δ*luxI_1_* cultures in final concentrations of 0.1, 0.5, 2.5, or 5 μM.

### Growth Measurements

For growth measurements strains were grown over night in 50 ml sea water medium in the dark. These pre-cultures were diluted with fresh medium to an initial OD_600_ of 0.01. For determination of the mutant’s growth in the presence of AHLs the respective volumes of the AHL stock solution (1 mM in DMSO) were added depending on the desired final AHL concentration. 200 μl of the culture were transferred into a Honeycomb 100-well plate and growth was monitored by half-hourly automatic OD_600_ measurements using the BioScreenC device (Oy Growth Curves Ab Ltd). Growth rates (μ) were determined for three biological replicates per treatment.

### Flow Cytometry

For flow cytometric analysis, samples at the selected cell density (OD_600_ 0.4) were fixed by addition of 2% glutaraldehyde. Samples were diluted 1000-folds in sterile filtered PBS buffer (pH 7.4) and DNA was stained with 10 μl per ml of sample of 100x SYBR Green I (Molecular Probes, Leiden, Netherlands). A minimum of 50 000 cells per sample, in at least two biological replicates, were analyzed using the FACS Canto flow cytometer (BD Bioscience). Fluorescence intensities were determined using the FITC filter with excitation at 488 nm and emission at 519 nm. Data were processed and analyzed using the “flowCore” (Hahne et al., 2009) package of the R BioConductor project.

### RNA Extraction and Labeling

Cells from at least two biological replicates per condition were harvested at mid-exponential growth phase (OD_600_ 0.4) by adding 4 ml culture to 800 μl stop-solution (5% phenol in ethanol) and centrifugation for 1 min at 13 000 rpm and 4°C. Cell pellets were immediately frozen in liquid nitrogen and stored at –70°C. Cell lysis was performed enzymatically for 25 min with TE buffer (pH 8.0) containing 15 mg/ml lysozyme followed by mechanical disruption using acid-washed glass beads and vortexing for 3 min. Lysates were applied to RNeasy spin columns (RNeasy Mini Kit, Qiagen, Germany) and RNA isolation was performed according to the manufacturer’s manual. In addition to the on-column digestion of genomic DNA a second DNase I-digestion was performed in solution, followed by a second purification and washing step with 80% ethanol. 2 μg of total RNA were labeled with the fluorescent dyes Cy3 and Cy5 using the USL Fluorescent Labeling Kit (Kreatech, Amsterdam, Netherlands) following the manufacturer’s protocol. 500 ng labeled RNA of each sample was fragmented and hybridized to a two-color Agilent Microarray applying a loop-design which allows a comparison of all samples with each other.

### Microarray Analysis

In this study a custom made Agilent microarray for *D. shibae* (AMADID 026232) has been used as previously described (Tomasch et al., 2011). Layout and probe annotation can be found in the gene expression omnibus (GEO) database^1^.

Gene expression in *D. shibae* Δ*luxI_1_* and *D. shibae* Δ*luxI_1_*-strain cultivated with synthetic AHLs was determined in comparison to the wild-type strain for at least two independent cultures at mid-exponential growth phase (OD_600_ 0.4). Microarray slides were scanned using the Agilent DNA microarray Scanner. Median spot intensities and background signals of the Cy3 and Cy5 channel were analyzed using the R environment^2^ and processed using the LIMMA package (Smyth, 2005). Background signals were subtracted using the “normexp” method (Ritchie et al., 2007), fluorescent signals were Loess normalized before quantile normalization was performed on all microarrays from one dataset. Signals from replicate probes for single genes were averaged. A linear model was fitted for each comparison as described (Smyth, 2004) to allow indirect comparisons across microarrays. The *p*-values were adjusted for false discovery rate (fdr) using the method described (Benjamini and Hochberg, 1995). Raw and processed data were deposited at the GEO database under the accession number GSE54420.

### Plasmid Conjugation

Three *D. shibae* DFL-12^T^ mutants with mariner transposon tagged plasmids (gentamicin resistance cassette) that were established in the recent large-scale mutagenesis approach (Ebert et al., 2013) were chosen as donor strains for plasmid conjugation. The flanking sequences of the transposons, which were determined via arbitrary PCR (O’Toole et al., 1999), are identical in both plasmids (NC_009955.1, NC_009957.1; Wagner-Döbler et al., 2010). Therefore it was initially unclear if the respective transposons (Tn) were located on the 191-kb plasmid pDSHI01 or the 126-kb plasmid pDSHI03. After detection of the plasmids with plasmid specific primers in *P. inhibens* DSM 17395 transconjugants, the exact integration site of the transposons was established. [Tn1, clone 40-A11: Dshi_3606/Dshi_3944 (integration position(s) 12584/13365), Tn2, clone 31-B1: Dshi_3624/Dshi_3962 (integration position(s) 26518/27299), Tn3, clone 11-D12: Dshi_3626/Dshi_3964 (integration position(s) 27457/28238)]. *P. inhibens* DSM 17395 was mutagenized with the EZ-Tn5 <R6Kγori/KAN-2> transposon kit (Epicenter; kanamycin resistance cassette). The genomic insertion sites of about 100 transposons were determined via arbitrary PCR (O’Toole et al., 1999; unpublished results) and one Tn-mutant whose chromosome was tagged in an intergenic region (integration position 521,196) was chosen as a recipient for the conjugation experiments.

Precultures of *D. shibae* donor strains and the *P. inhibens* recipient were cultivated at 28°C in test tubes with MB medium containing the antibiotics gentamicin (40 μg/ml) and kanamycin (120 μg/ml), respectively. 1000 μl of *D. shibae* and 100 μl of *P. inhibens* cells that were grown to the exponential phase were added to a new test tube with 3.5 ml MB medium and incubated overnight with gentle shaking (50 rpm). The conjugation cocktail was plated on 0.5x MB agar plates with both antibiotics and incubated for 3 days at 28°C. Transconjugants were picked, incubated in 0.5x MB medium for 3 days and plated on 0.5x MB agar plates under continuous antibiotic selection. Passaging of single colonies was repeated twice in order to obtain authentic transconjugants without non-conjugated *P. inhibens* recipient and residual *D. shibae* donor cells.

### PCR-based Differentiation of *P. inhibens* Transconjugants

Plasmid DNA of the transconjugants was isolated with the NucleoSpin Plasmid DNA kit (Macherey-Nagel) and used for PCR amplification with the Crimpson Taq DNA polymerase. Two specific primer sets were established for the 191-kb as well as the 126-kb plasmid that allow the differentiation between the two sister plasmids: PCR-A pDSHI01 (191-kb), P450 (5′-TTACGAAAAACCCGCAGAAGG-3′), and P451 (5′-CCGTTGACCCTTTCGTGGCTG-3′); PCR-B pDSHI01 (191-kb), P430 (5′-TCTGGCTGCGTGGTGGCTTTC-3′), and P431 (5′-TGCGCTATAGTGCTCTCAACA-3′); PCR-C pDSHI03 (126-kb), P432 (5′-GGCACCATCGTCGGAACCAAT-3′), and P433 (5′-TGGTATCAGGCATTCGCTTCA-3′); PCR-D pDSHI03 (126-kb), P059 (5′-CTGACCGTGTTGGAAAGAAGT-3′), and P064 (5′-GCACGAAAAGGCAAAAGA-3′). PCR amplification of the 262-kb plasmid of *P. inhibens* with P100 (5′-AAACCTTCGTGCCGCTTGTGA-3′), and P105 (5′-CCCAGTTGGAGGATGAGG-3′) served as a positive control (PCR-E). PCR reactions A, B, C, and E were performed with 1 ng of purified plasmid DNA, 5 ng were used for reaction D.

### Pulsed-Field Gelelectrophoresis Analysis of Transconjugants

The plasmid composition of the kanamycin-tagged *P. inhibens* DSM 17395 transconjugants was determined with one-dimensional pulsed-field gel electrophoresis (PFGE) as previously described (Pradella et al., 2010). The completely sequenced strains *D. shibae* DSM 16493^T^ and *P. inhibens* DSM 17395, which served as donor, and recipient for plasmid conjugation, were used as references (Wagner-Döbler et al., 2010; Thole et al., 2012).

## Author Contributions

JP, DP, JT, PT, and IW-D designed the study. DP, VM, OP, and ME performed the experiments. DJ provided materials. DP, JP, JT, DJ, and IW-D analyzed the data and wrote the manuscript.

## Conflict of Interest Statement

The authors declare that the research was conducted in the absence of any commercial or financial relationships that could be construed as a potential conflict of interest.

## Footnotes

1 http://www.ncbi.nlm.nih.gov/geo/query/acc.cgi?acc=GPL11243

2 https://www.r-project.org/
