# Supplementary material for: Gene Flow Across Genus Barriers – Conjugation of Dinoroseobacter shibae’s 191-kb Killer Plasmid into Phaeobacter inhibens and AHL-mediated Expression of Type IV Secretion Systems
Source: Front Microbiol. 2016 May 31;7:742. doi: 10.3389/fmicb.2016.00742 (PMC4886583; doi:10.3389/fmicb.2016.00742)
Supplement: Supplementary file 2 [file Image_1.PDF]

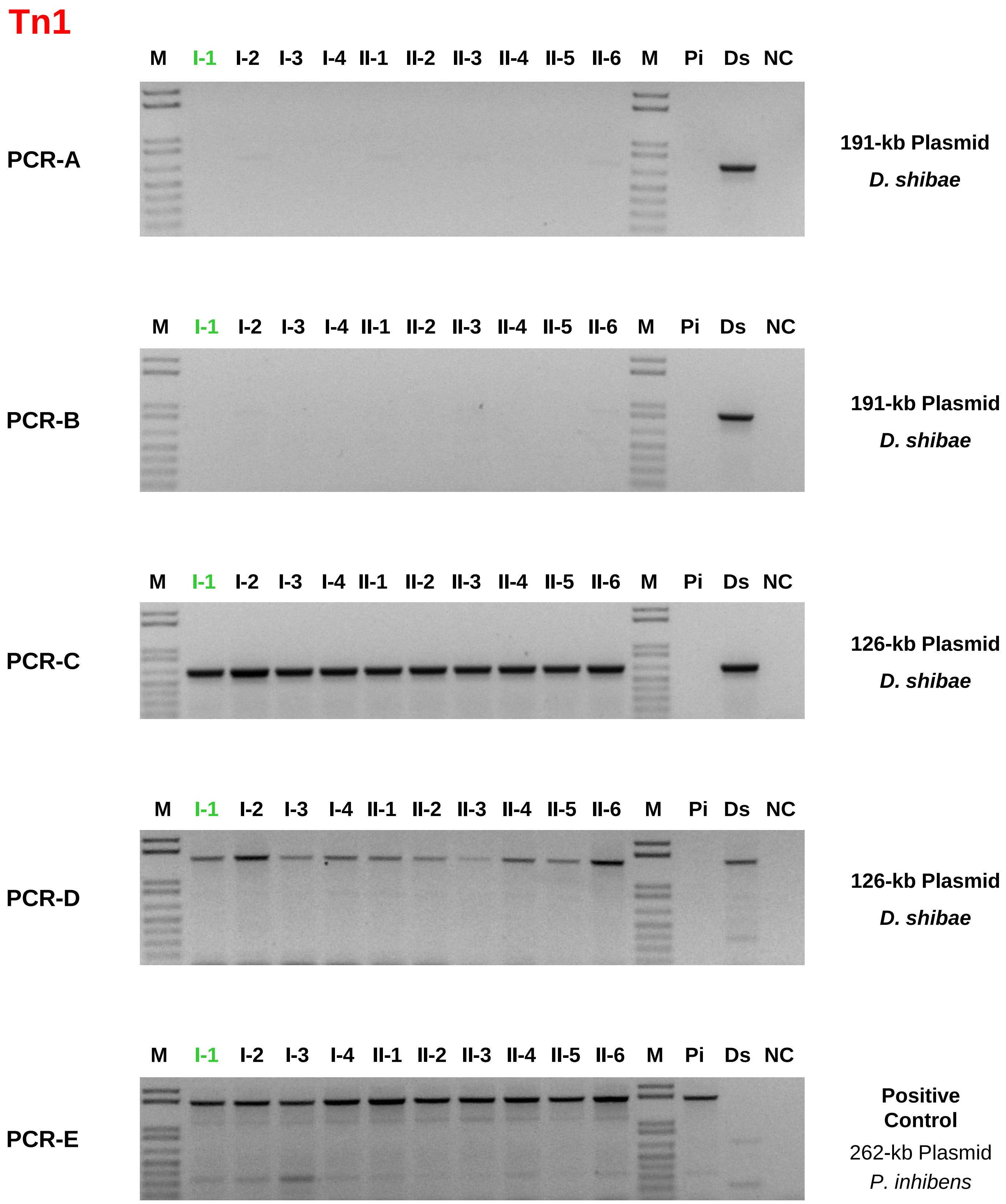


**Suppl. Fig. S2a: PCR-test of *Phaeobacter inhibens* transconjugants.** The transposon **Tn1** (clone 40-A11) was inserted into the cation diffusion facilitator family transporter gene of the 191-kb (Dshi_3606) or the 126-kb plasmid (Dshi_3944) of *Dinoroseobacter shibae* (Ebert et al. 2013). PCR reactions were performed with specific primers against the 191-kb / 126-kb plasmid of *D. shibae* and the 262-kb plasmid of *P. inhibens*. Transconjugants whose plasmid profile was determined with PFGE are highlighted in green. I, II, conjugation experiment I and II; Pi, control PCR with *P. inhibens* DNA; Ds, control PCR with *D. shibae* DNA; NC, negative control.

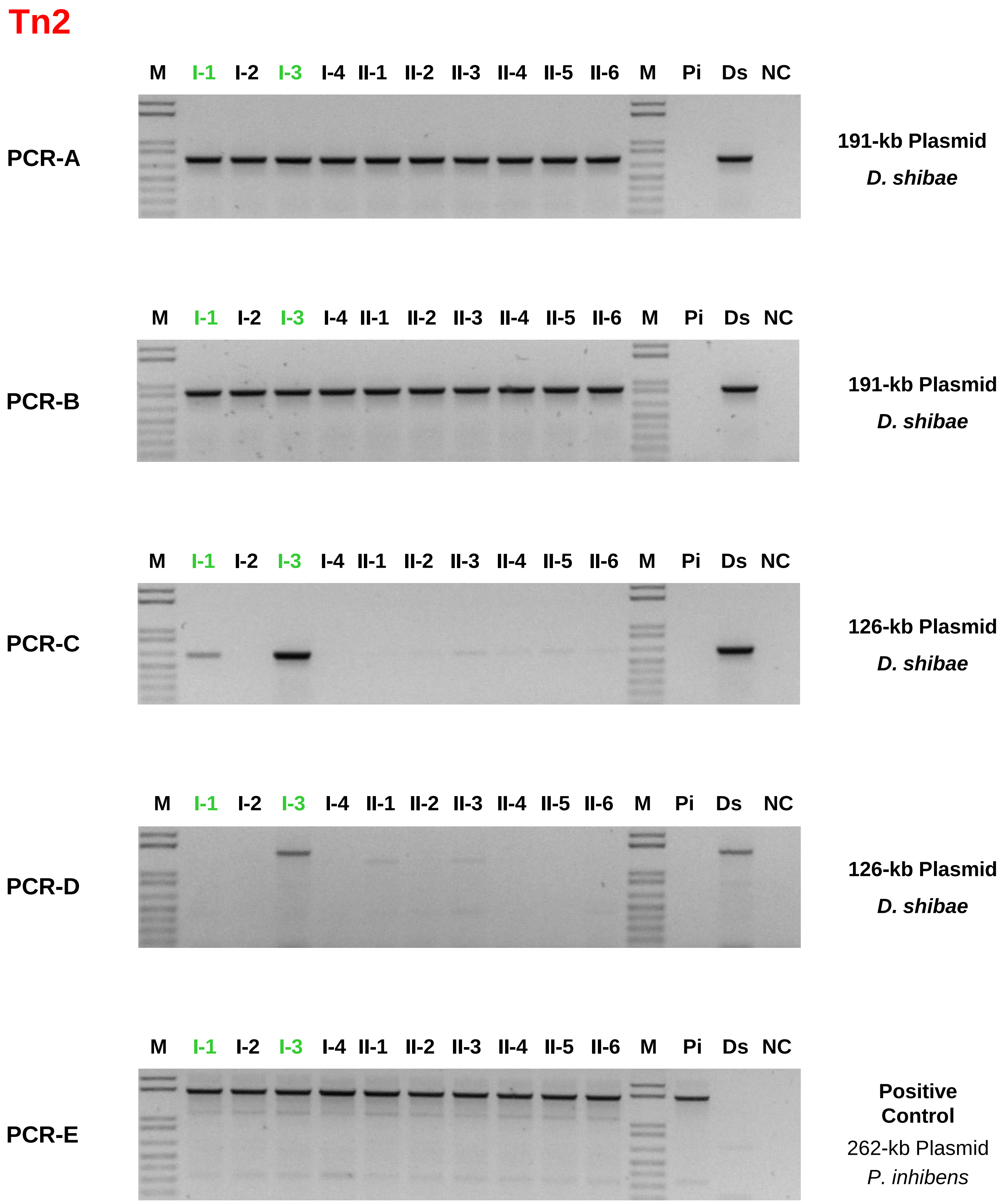


**Suppl. Fig. S2b: PCR-test of *Phaeobacter inhibens* transconjugants.** The transposon **Tn2** (clone 31-B1) was inserted into the cation diffusion facilitator family transporter gene of the 191-kb (Dshi_3624) or the 126-kb plasmid (Dshi_3962) of *Dinoroseobacter shibae* (Ebert et al. 2013). PCR reactions were performed with specific primers against the 191-kb / 126-kb plasmid of *D. shibae* and the 262-kb plasmid of *P. inhibens*. Transconjugants whose plasmid profile was determined with PFGE are highlighted in green. I, II, conjugation experiment I and II; Pi, control PCR with *P. inhibens* DNA; Ds, control PCR with *D. shibae* DNA; NC, negative control.

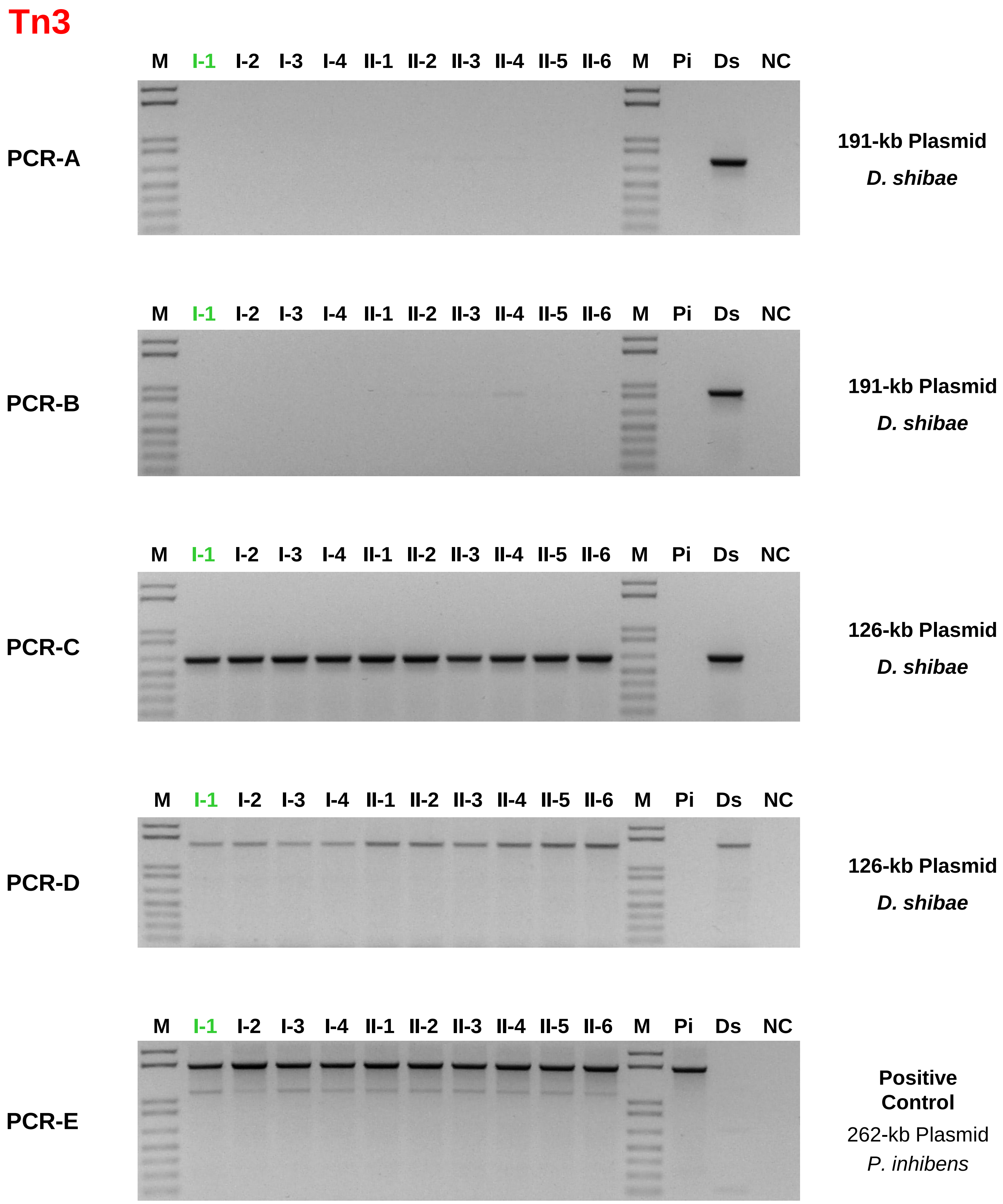


**Suppl. Fig. S2c: PCR-test of *Phaeobacter inhibens* transconjugants.** The transposon **Tn3** (clone 11-D12) was inserted into the cation diffusion facilitator family transporter gene of the 191-kb (Dshi_3626) or the 126-kb plasmid (Dshi_3964) of *Dinoroseobacter shibae* (Ebert et al. 2013). PCR reactions were performed with specific primers against the 191-kb / 126-kb plasmid of *D. shibae* and the 262-kb plasmid of *P. inhibens*. Transconjugants whose plasmid profile was determined with PFGE are highlighted in green. I, II, conjugation experiment I and II; Pi, control PCR with *P. inhibens* DNA; Ds, control PCR with *D. shibae* DNA; NC, negative control.
