# Supplementary material for: Gene Flow Across Genus Barriers – Conjugation of Dinoroseobacter shibae’s 191-kb Killer Plasmid into Phaeobacter inhibens and AHL-mediated Expression of Type IV Secretion Systems
Source: Front Microbiol. 2016 May 31;7:742. doi: 10.3389/fmicb.2016.00742 (PMC4886583; doi:10.3389/fmicb.2016.00742)
Supplement: Supplementary file 3 [file Image_2.PDF]

Transposon Insertion Sites in *Dinoroseobacter shibae* Plasmids (191kb, 126kb)

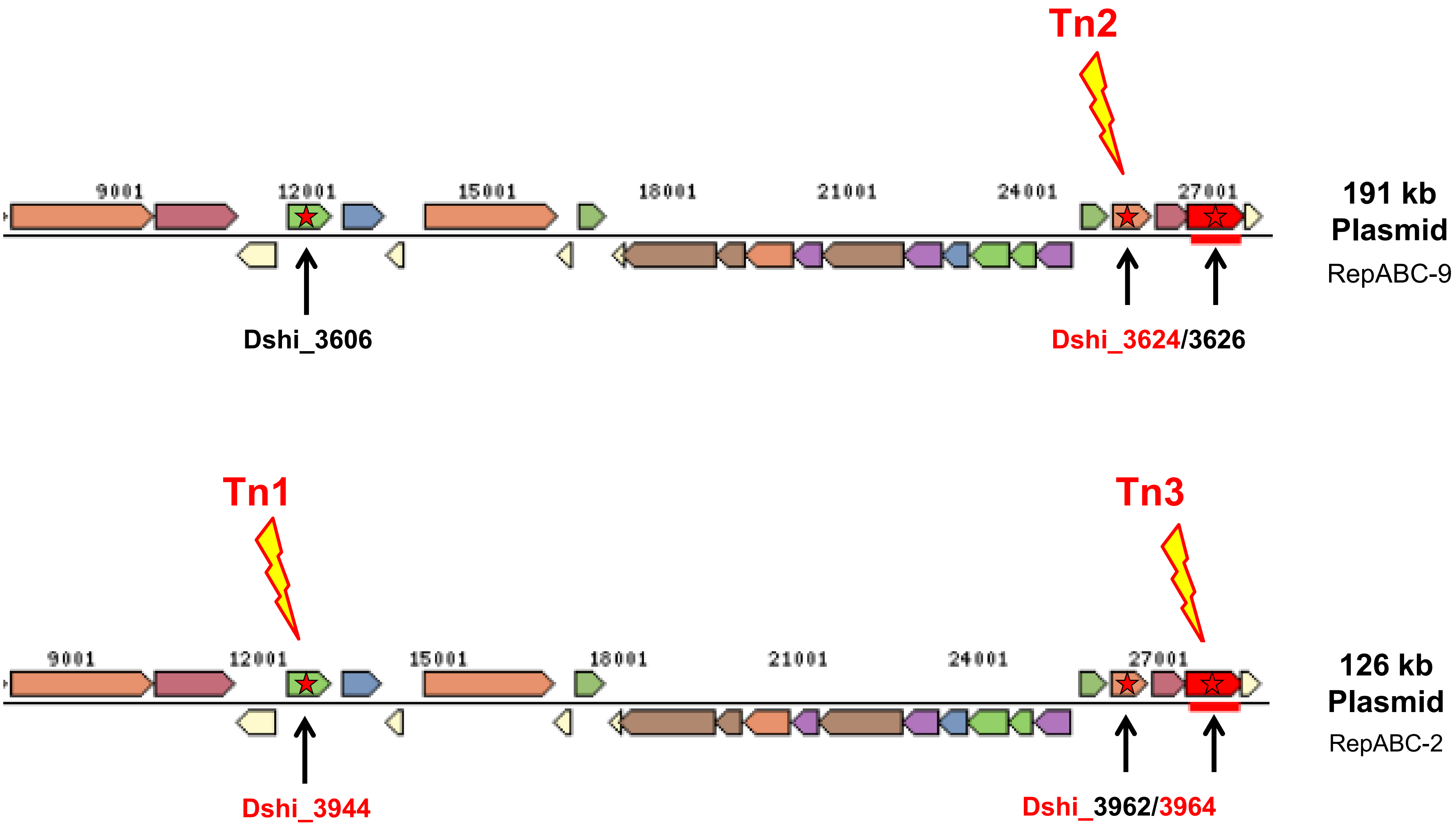

Tn1: Dshi_3606 versus Dshi_3944 - Cytochrome C biogenesis protein (40-A11)

Tn2: Dshi_3624 versus Dshi_3962 - Cation efflux protein (31-B1)

Tn3: Dshi_3626 versus Dshi_3964 - Cation efflux protein (11-D12)
