# Supplementary material for: Gene Flow Across Genus Barriers – Conjugation of Dinoroseobacter shibae’s 191-kb Killer Plasmid into Phaeobacter inhibens and AHL-mediated Expression of Type IV Secretion Systems
Source: Front Microbiol. 2016 May 31;7:742. doi: 10.3389/fmicb.2016.00742 (PMC4886583; doi:10.3389/fmicb.2016.00742)
Supplement: Supplementary file 4 [file Image_3.PDF]

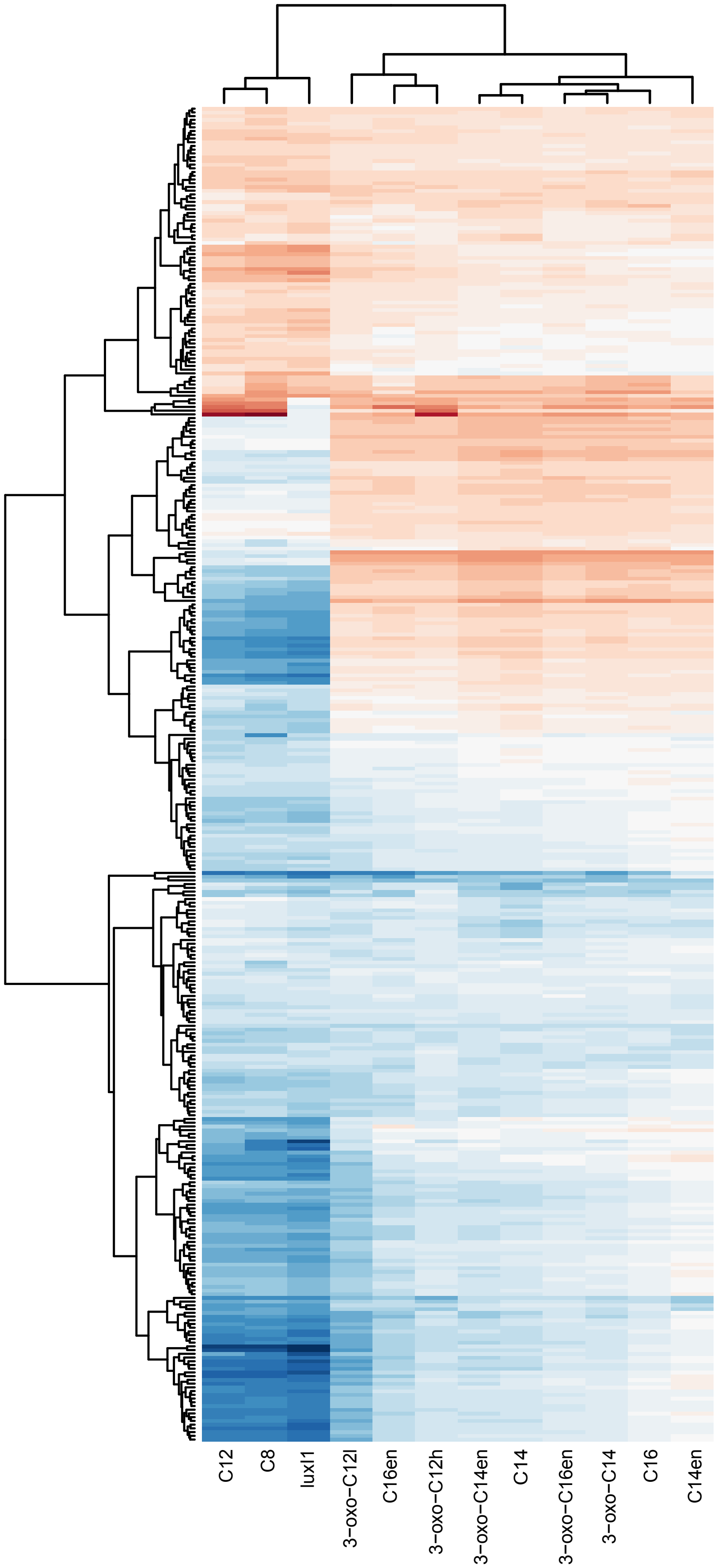

Figure S1: **Heatmap visualization of log2 fold changes of differentially regulated genes in Δ*luxI1* and Δ*luxI1* supplemented with the indicated AHLs in comparison to the wild-type**. Genes with a log2 change above 1 and an adjusted p-value below 0.01 in at least one sample were considered as differentially regulated genes. Log2 fold changes of these 360 genes are visualized comparing the quorum sensing mutant Δ*luxI1* alone and Δ*luxI1* supplemented with the indicated AHLs to the wild-type.
